# Clinical outcomes of endoscopic submucosa dissection for high-grade dysplasia from endoscopic forceps biopsy

**DOI:** 10.1007/s10120-016-0665-6

**Published:** 2016-11-07

**Authors:** Dae Gon Ryu, Cheol Woong Choi, Dae Hwan Kang, Hyung Wook Kim, Su Bum Park, Su Jin Kim, Hyeong Seok Nam

**Affiliations:** Department of Internal Medicine, Medical Research Institute, Pusan National University School of Medicine and Research Institute for Convergence of Biomedical Science and Technology, Pusan National University Yangsan Hospital, Beomeo-ri Mulgeum-eup, Yangsan-si, Gyeongsangnam-do 626-770 Korea

**Keywords:** Early gastric cancer, Dysplasia, Endoscopic submucosal dissection, Biopsy

## Abstract

**Background and study aims:**

Although the Vienna Classification recommends endoscopic resection for gastric high-grade dysplasia (HGD), many resected lesions are diagnosed as gastric cancer after endoscopic resection. This study aims to evaluate the clinical outcomes of gastric HGD identified by endoscopic forceps biopsy (EFB) after endoscopic submucosal dissection (ESD) and factors associated with discrepant results.

**Patients and methods:**

From December 2008 to July 2015, a total of 427 lesions diagnosed as initial HGD by EFB were enrolled. The rate of early gastric cancer (EGC) and factors predicting diagnosis upgrade were analyzed retrospectively.

**Results:**

Tumors ranged between 2 and 65 mm in size (median 12.59). En bloc and complete resection rates were 97.4 and 95.3%, respectively. The diagnostic discrepancy rate was 76.3%. Upgrade and downgrade rates of pathological diagnoses were 66.5 and 9.8%, respectively. Central depression (OR 4.151), nodular surface (OR 5.582), surface redness (OR 2.926), lesion location (upper third of the stomach) (OR 3.894), and tumor size ≥10 mm (OR 2.287) were significantly associated with EGC. Nodular surface (OR 2.746), submucosal fibrosis (OR 3.958), lesion location (upper third of the stomach) (OR 6.652), and tumor size ≥10 mm (OR 4.935) significantly predicted invasive submucosal cancer.

**Conclusions:**

Central depression, nodular surface, surface redness, lesion location, large tumor size, and submucosal fibrosis were associated with EGC or submucosal cancer. Caution must be used in treating lesions with these features with ESD.

## Introduction

In recent years, endoscopic submucosal dissection (ESD) has become an accepted curative treatment modality for the treatment of high-grade dysplasia (HGD) or early gastric cancer (EGC) without lymph node metastasis. ESD is preferred because it is less invasive and expensive and results in a better quality of life compared with surgical gastric resection. In order to treat EGC by ESD, early detection of EGC or dysplastic lesions is essential, especially in countries where gastric cancer is highly prevalent. In South Korea, the National Cancer Screening Program is in operation and biennial esophago-gastro-duodenal endoscopy is recommended for men and women over 40 years old. With the widespread availability of screening endoscopy, early detection of precancerous lesions and EGC has increased. In South Korea, the proportion of stage IA patients has increased by ~57% during the last 10 years [1, 2].

Gastric adenoma/dysplasia is regarded as a precancerous lesion. The risk of carcinoma generally increases with the histological grade of the dysplasia (low to high grade) [3]. Gastric HGD (category 4 in the Vienna Classification) is highly predictive of invasive carcinoma, which either coexists or appears within a short time after biopsy. Therefore, from the revised Vienna Classification, HGD should be removed by endoscopic resection [4]. Although an endoscopic forceps biopsy (EFB) is the best method to diagnose EGC, the misdiagnosis rate of EFB specimens for gastric superficial neoplasm is reported to be up to 40–55% [5, 6]. If the pathologic result after ESD subsequently shows gastric cancer, additional surgical treatment may be considered according to the pathological type (well vs. poorly differentiated adenocarcinoma), depth of invasion (mucosal cancer or submucosal invasive cancer or lymphatic invasion), or resection margin status (free resection margin vs. involved resection margin). However, there is no way to determine the submucosal or lymphatic invasion accurately before endoscopic resection.

In the present study, we retrospectively evaluated the final ESD outcomes of gastric HGD from EFB and analyzed the endoscopic characteristics associated with invasive EGC after ESD.

## Materials and methods

### Patients

From December 2008 to July 2015, the medical records of patients who were diagnosed with gastric HGD at the Pusan National University Yangsan Hospital, South Korea, were retrospectively reviewed. For patients diagnosed with HGD at another institution and referred to our hospital for treatment, endoscopic biopsy was performed again, and tissue samples were re-analyzed.

In principle, endoscopic resection is recommended for patients with a diagnosis of HGD. Among our study group, two patients did not undergo endoscopic resection during the study period because of underlying liver cirrhosis and hepatocellular carcinoma, and an expected poor life expectancy, although the patients continued to undergo endoscopic assessment every 6 months for observation of disease progression. Another three patients underwent direct surgery despite confirmation of a diagnosis of HGD on re-biopsy for the following reasons: One patient presented with a discursive ulcer, with a diagnosis of mucosal cancer confirmed on surgical intervention. For another patient, the lesion enclosed the whole cardia, with a diagnosis of invasive submucosal (SM) cancer without lymph node metastasis. The third patient who underwent direct surgery presented with a lesion with involvement from the pyloric ring to the duodenum bulb, with three additional adenomas in the antrum, conditions that increase the risk for stenosis post-endoscopy. Once this was explained to the patient, the patient accepted direct surgery, with the diagnosis of HGD being confirmed with surgery.

**Fig. 1:**
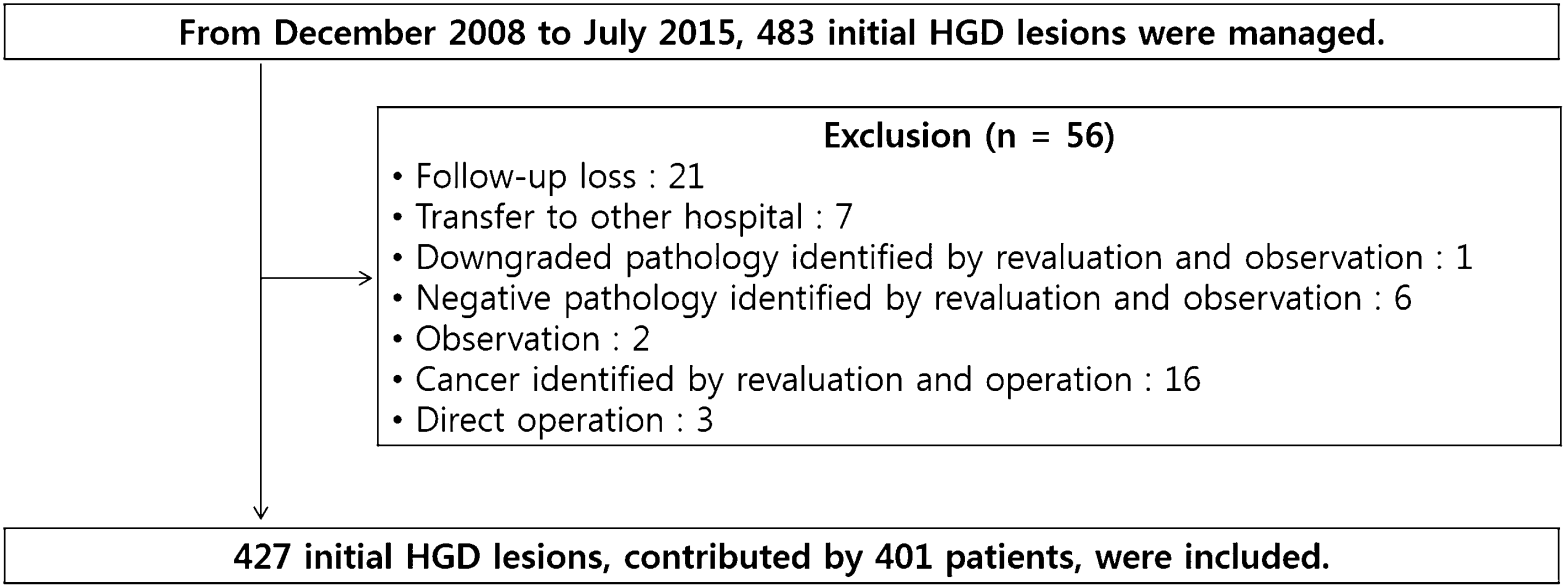
Flow chart of the enrolled lesions in this study. *ESD* endoscopic submucosal dissection, *EFB* endoscopic forceps biopsy, *HGD* high-grade dysplasia

During the study period, 483 initial HGD lesions, contributed by 457 patients, were managed by endoscopic resection. Among these, 21 patients were lost to follow-up, and 7 patients were transferred to another medical institution. In one patient, the pathological diagnosis of the lesion was downgraded to low-grade dysplasia (LGD) at the time of re-biopsy, followed by a 9-month follow-up period of observation without further intervention. A negative pathological report was also provided for another six lesions at the time of re-biopsy, with three of these patients being lost to follow-up after the negative diagnosis, while the remaining three patients were followed up by observation. Ultimately, the data from 427 initial HGD lesions, contributed by 401 patients, were included in our analysis (Fig. 1).

Written informed consent was obtained from all patients prior to the procedure. The study was approved by the ethics committee of the Institutional Review Board (Institutional Review Board no. 05-2016-040).

### Endoscopic biopsy

Diagnostic endoscopy (using GIF-H260 or GIF-H290; Olympus Optical Co., Ltd., Tokyo, Japan) and EFB were performed in all patients before ESD. Most of the patients were referred from other hospitals and underwent additional EFB or review of referred biopsy specimens.

### ESD procedure

We performed ESD using the previously described technique [5]: after lesion marking, normal saline with an epinephrine and indigocarmine mixture was injected into the submucosal layer to elevate the lesion from the muscularis propria. The mucosa surrounding the lesion was then precut using an electrosurgical generator (ERBE VIO 300D, Endocut I mode, Effect 3, duration 2; Erbe Co, Tubingen, Germany) with a flex knife, dual-knife, or an insulation-tipped electrosurgical knife 2 (IT 2); lastly, the connective tissue of the submucosa beneath the lesion was dissected with a coagulation current (Swift coagulation 60 W, ERBE VIO 300D). After removal of the lesions, preventive post-ESD coagulation was performed for all visibly exposed vessels with hot biopsy forceps.

### Endoscopic and pathologic evaluation

Baseline characteristics and endoscopic findings of all enrolled lesions were assessed. Endoscopic photographs and endoscopic reports were reviewed to determine the features of the lesions. All endoscopic diagnoses were performed by two endoscopists (DG Ryu, MD, and SJ Kim, MD), both of whom had received training on reviewing approximately 100 typical endoscopic findings prior to assessment of the endoscopic biopsy images. All reviews were performed in a blinded fashion. The diagnosis was consistent between the two endoscopists for 382 of the 427 lesions. For the remaining 45 lesions, the diagnosis was attained by discussion and consensus. The Paris Classification was used to define the gross types of superficial lesions, which were divided into elevated, flat, or depressed [7]. Central depression, surface redness, nodularity, ulceration, and submucosa fibrosis were also evaluated. Central depression was defined as the inner part of the lesion being depressed compared to the surrounding, regardless of gross type. Surface redness was defined as a red discoloration on the mucosal surface of the lesion compared to the surrounding mucosa. Surface nodularity was defined as the presence of irregularly raised or nodular mucosa. Lesions with ulcerations or scarring from previous ulceration (converging folds or deformity of the muscularis propria or fibrosis in the submucosa) were regarded as ulcerated. If submucosa fibrosis was observed during the ESD procedure, this was recorded with endoscopic pictures. The location of lesions was described using the Japanese Classification of Gastric Cancer [8]. In this system, the gastric area is divided into three equal sections: the upper, middle, and lower thirds of the stomach.

All of the endoscopically resected tissue slides were blindly reviewed by two pathologists. Discordant cases were re-evaluated under multi-headed microscope to reach agreement. The resected specimens were stretched, pinned, and fixed with formalin. Piecemeal-resected specimens were reconstructed as much as possible. The fixed specimen was sectioned at 2-mm intervals. All of the lesions were measured on the length of the major and minor axes. All of the lesions were classified as gastrointestinal epithelial neoplasia according to the Vienna Classification [4].

### Statistical analysis

Data were analyzed based on individual lesions because some patients had multiple lesions. Univariate analysis with chi-square test or Fisher’s exact test for categorical variables and Student’s *t* test for continuous variables were performed. Multivariate analysis with a multiple logistic regression model was performed to identify risk factors for EGC and furthermore submucosal or lymphovascular invasive cancer. *P* < 0.05 was considered statistically significant. Statistical calculations were performed with SPSS version 21.0 for Windows (SPSS Inc., Chicago, IL, USA).

## Results

**Fig. 2:**
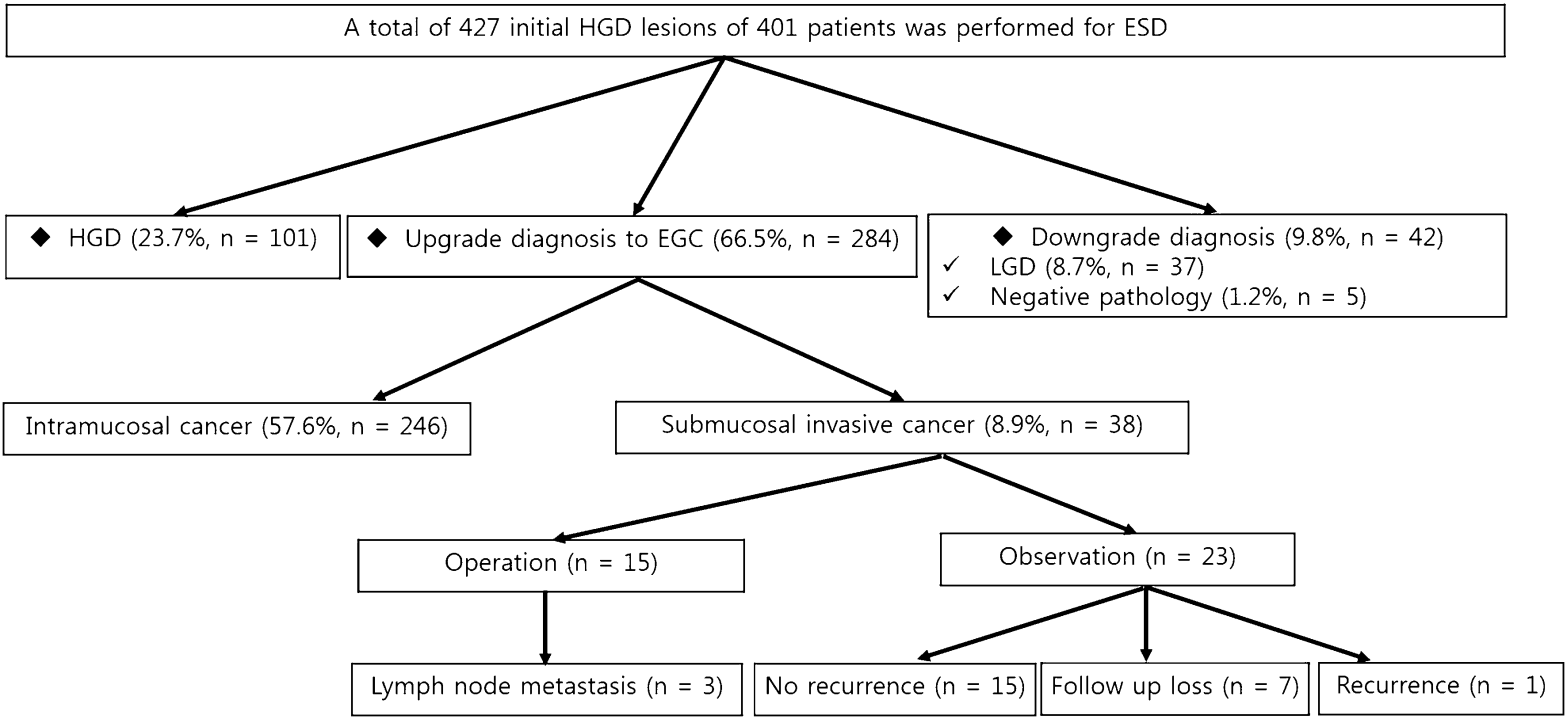
Final results of gastric high-grade dysplasia from endoscopic forceps biopsy. *ESD* endoscopic submucosal dissection, *EFB* endoscopic forceps biopsy, *HGD* high-grade dysplasia, *EGC* early gastric cancer

The patients’ mean age was 63.89 ± 8.53 years. The range of tumor size was 2–65 mm (median 12.59). The main location of the lesions was the lower third of the stomach (Table 1). En bloc resection and complete resection rates were 97.4% (416/427) and 95.3% (407/427). The diagnostic discrepancy rate was 76.3% (326/427). The up- and downgrade rates of the pathological diagnosis were 66.5% (284/427) and 9.8% (42/427), respectively. Among those undergoing EGC after ESD, 38 lesions were found to be submucosal invasive lesions (including 6 with lymphovascular invasion). Among the submucosal invasive lesions, 15 patients underwent additional operations, and lymph node metastasis was found in 3 patients (Fig. 2).

**Table 1:** Baseline characteristics

	Total (*n* = 427)
Mean age [years (±SD)]	63.89 ± 8.53
En-bloc resection, *n* (%)	416 (97.4)
Complete resection, *n* (%)	407 (95.3)
Male, *n* (%)	337 (78.9)
Helicobacter pylori infection, *n* (%)	248 (58.1)
Gross type, *n* (%)	
Elevated	127 (29.7)
Flat	133 (31.1)
Depressed	167 (39.1)
Central depression, *n* (%)	212 (49.6)
Nodular surface, *n* (%)	176 (41.2)
Surface redness, *n* (%)	215 (50.4)
Tumor location, *n* (%)	
Upper third of stomach	26 (6.1)
Middle third of stomach	42 (9.8)
Lower third of stomach	359 (84.1)
Tumor size, *n* (%)	
≤10 mm	198 (46.4)
>10 mm	229 (53.6)
Range, mean size (mm)	2–65 (12.59)

**Fig. 3:**
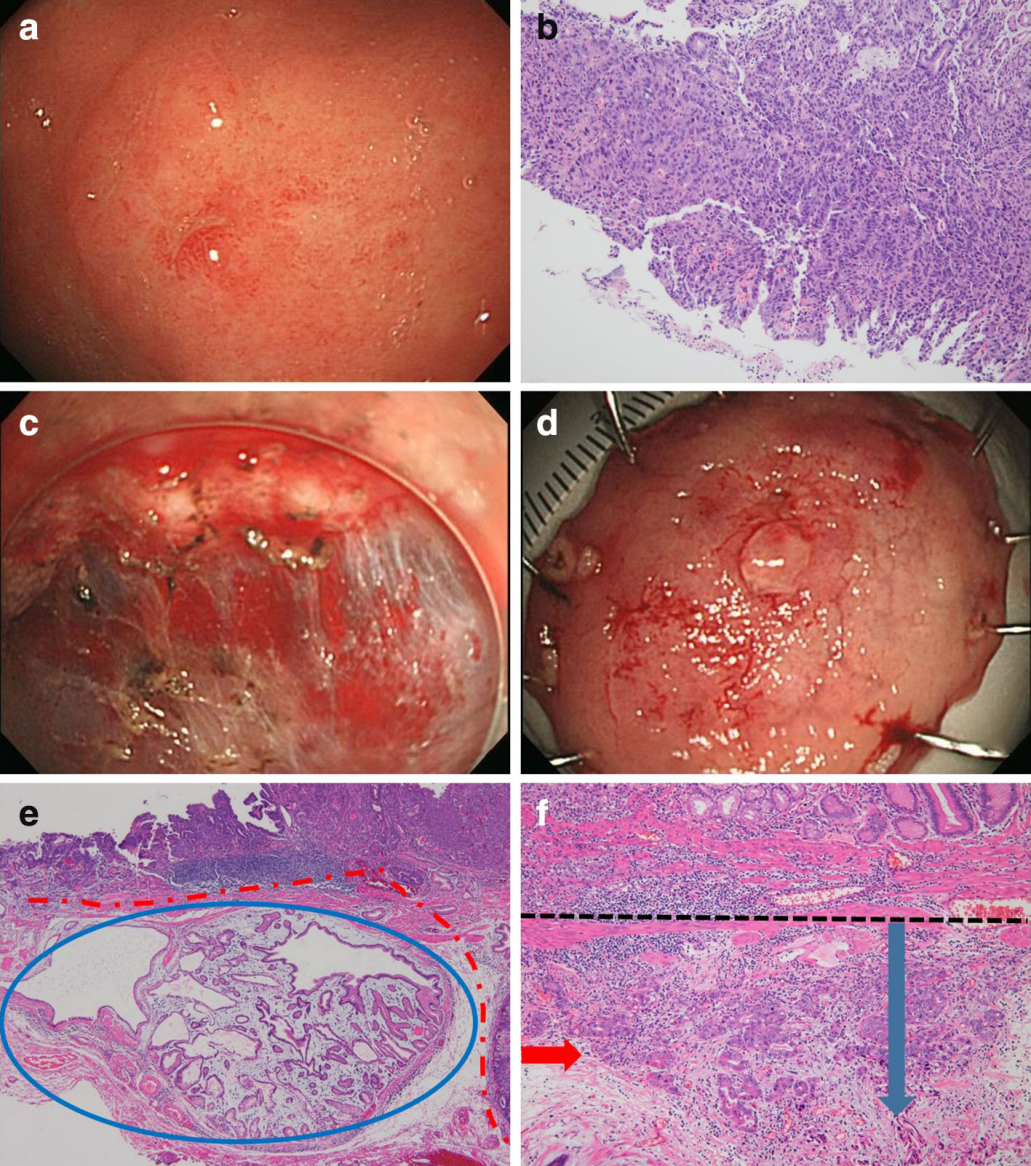
A case of upgrade diagnosis to submucosal invasive cancer. **a** Conventional endoscopic image: the lesion located at the mid-body lesser curvature with redness, nodular and central depressed feature. **b** Histology of EFB shows tubular adenoma with HGD. **c** Endoscopic finding during endoscopic submucosal dissection. **d** En-block resected ESD specimen (long diameter 4.3 cm). **e** Pathologically diagnosed with adenocarcinoma (*above red dash*) and normal gland is seen pressed down below the submucosa (*in blue oval line*). **f** H&E stain, ×100 magnification: It seems that the cancer (*red arrow*) has invaded the submucosa about 2000 μm (*black dash* muscular mucosa, *blue arrow* submucosa). *ESD* endoscopic submucosal dissection, *EFB* endoscopic forceps biopsy, *HGD* high-grade dysplasia

Endoscopic characteristics associated with EGC were analyzed (Table 2). Multivariate analysis revealed that central depression [OR 4.151 (95% CI 2.340–7.363)], nodular surface [OR 5.582 (95% CI 3.230–9.649)], surface redness [OR 2.926 (95% CI 1.747–4.901)], lesion location (upper third of the stomach) [OR 3.894 (95% CI 1.110–13.666)], and larger tumor size (≥10 mm) [OR 2.287 (95% CI 1.387–3.770)] were significantly associated with EGC. Multivariate analysis revealed that surface nodularity [OR 2.746 (95% CI 1.246–6.053)], submucosal fibrosis [OR 3.958 (95% CI 1.822–8.596)], lesion location (upper third of the stomach) [OR 13.051 (95% CI 3.229–52.754)], and larger tumor size (≥10 mm) [OR 6.652 (95% CI 2.357–18.772)] were significant factors associated with submucosal invasive cancer (Table 3). A case of diagnosis upgraded to deep submucosal invasive cancer after ESD is illustrated in Fig. 3.

**Table 2:** Characteristics and associated risk factors for upgrade diagnosis HGD to EGC in univariate and multivariate analysis (non-cancer, *n* = 143/EGC, *n* = 284)

Variables	Non-cancer, *n* (%)	EGC, *n* (%)	Univariate analysis			Multivariate analysis		
			OR	95% CI	*P* value	OR	95% CI	*P* value
Age >60	86 (60.1)	196 (69.0)	1.476	0.971–2.244	0.068	1.477	0.887–2.457	0.134
Mean age [year ± (SD)]	62.69 ± 8.74	64.66 ± 8.32						
Male gender	106 (74.1)	231 (81.3)	1.521	0.943–2.455	0.085	1.164	0.631–2.147	0.628
HP infection	81 (56.6)	167 (58.8)	1.093	0.728–1.640	0.670	1.082	0.657–1.783	0.758
Gross type								
Elevated (ref.)	57 (39.9)	70 (24.6)	1.000			1.000		
Flat	49 (34.3)	84 (29.6)	1.396	0.850–2.293	0.187	1.031	0.531–2.003	0.928
Depressed	37 (25.9)	130 (45.8)	2.861	1.726–4.744	<0.001	1.975	1.001–3.897	0.050
Central depression	39 (27.3)	173 (60.9)	4.156	2.681–6.443	<0.001	4.151	2.340–7.363	<0.001
Nodular surface	26 (18.2)	150 (52.8)	5.123	3.157–8.313	<0.001	5.582	3.230–9.649	<0.001
Surface redness	51 (35.7)	164 (52.8)	2.465	1.627–3.735	<0.001	2.926	1.747–4.901	<0.001
Ulcer	27 (18.9)	46 (16.2)	0.830	0.491–1.403	0.487	0.358	0.184–0.694	0.002
SM fibrosis	22 (15.4)	70 (24.6)	1.799	1.061–3.052	0.028	1.238	0.655–2.339	0.511
Tumor location								
Lower (ref.)	127 (88.8)	232 (81.7)	1.000			1.000		
Upper	4 (2.8)	22 (7.7)	3.011	1.015–8.929	0.038	3.894	1.110–13.666	0.034
Middle	12 (8.4)	30 (10.6)	1.369	0.677–2.766	0.381	1.604	0.714–3.601	0.252
Size >10 mm	58 (40.6)	171 (60.2)	2.218	1.472–3.341	<0.001	2.287	1.387–3.770	0.001
Mean size [mm ± (SD)]	10.52 ± 7.30	13.01 ± 8.39						

HGD, high-grade dysplasia; EGC, early gastric cancer; SM, submucosa; HP, *Helicobacter pylori*

**Table 3:** Risk factors associated with upgrade diagnosis HGD to SM or LV invasive cancer in univariate and multivariate analysis (reference, *n* = 389/SM or LV invasive cancer, *n* = 38)

Variables	Reference, *n* (%)	SM cancer, *n* (%)	Univariate analysis			Multivariate analysis		
			OR	95% CI	*P* value	OR	95% CI	*P* value
Age >60	256 (65.8)	26 (68.4)	1.126	0.550–2.302	0.746	0.986	0.427–2.276	0.974
Mean age [year ± (SD)]	63.78 ± 8.32	65.19 ± 10.91						
Male gender	308 (79.2)	29 (76.3)	0.847	0.386–1.861	0.680	0.529	0.209–1.342	0.180
HP infection	225 (57.8)	23 (60.5)	1.118	0.566–2.208	0.749	1.203	0.531–2.729	0.658
Gross type								
Elevated (ref.)	112 (28.8)	15 (39.5)	1.000			1.000		
Flat	126 (32.4)	7 (18.4)	0.415	0.163–1.054	0.058	0.443	0.150–1.314	0.142
Depressed	151 (38.8)	16 (42.1)	0.791	0.375–1.668	0.537	0.599	0.237–1.512	0.278
Central depression	189 (48.6)	23 (60.5)	1.623	0.822–-3.203	0.160	1.454	0.601-3.518	0.407
Nodular surface	153 (39.3)	23 (60.5)	2.365	1.196–4.676	0.011	2.746	1.246–6.053	0.012
Surface redness	190 (48.8)	25 (65.8)	2.014	1.001–4.052	0.046	1.974	0.890–4.379	0.094
Ulcer	64 (16.5)	9 (23.7)	1.576	0.712–3.3488	0.258	1.449	0.276–7.593	0.661
SM fibrosis	73 (18.8)	19 (50.0)	4.329	2.182–8.587	<0.001	3.958	1.822–8.596	0.001
Tumor location								
Lower (ref.)	337 (86.6)	22 (57.9)	1.000			1.000		
Upper	17 (4.4)	9 (23.7)	8.110	3.245–20.265	<0.001	6.652	2.357–18.772	<0.001
Middle	35 (9.0)	7 (18.4)	3.064	1.222–7.680	0.013	2.118	0.766–5.854	0.148
Tumor size >10 mm	196 (50.4)	33 (86.8)	6.499	2.485–16.996	<0.001	4.935	1.813–13.428	0.002
Mean size [mm ± (SD)]	11.86 ± 7.20	20.03 ± 11.65						

HGD, high-grade dysplasia; SM, submucosa; LV, lymphovascular; HP, *Helicobacter pylori*

On follow-up at 3–6 months after endoscopic resection, a *Helicobacter pylori* (HP) test (rapid urease test or biopsy or blood antibody test or urea breath test) was performed in all patients. Absence of infection was defined by two consecutive negative HP test results, with one positive result being considered as a case of infection. We identified an incidence rate of HP infection of 58.1% (248/427 cases). Among patients with no HP infection, 35.8% (64/179) of patients had received prior HP treatment. However, there was no difference in the rate of HP infection between the final HGD group and EGC group. Moreover, there was no difference in HP infection rate between the final HGD group and SM invasive cancer group (Tables 2, 3).

## Discussion

Since ESD has been widely implemented for the treatment of EGC, early detection of precancerous lesions such as HGD has been more important. In recent years, more than 50% of gastric cancers have been detected as EGC in South Korea [1, 2]. Although EFB can be used as a basic diagnostic tool for the initial treatment of gastric superficial neoplasm, gastric HGD (category 4 in the Vienna Classification) is shown to be cancer in about 27.6–80% of cases after endoscopic resection [6, 9, 10]. The possible reasons for this discrepancy may be as follows. First, forceps biopsy samples are small and do not represent the entire lesion. Second, cancer sometimes exists as hidden foci in other parts of the lesion. Third, the atypia of adenoma and adenocarcinoma is too subtle to detect in a small biopsy specimen [6]. Fourth, accurate targeted biopsy through EFB can be difficult because of the location of lesions. In the present study, 66.5% (284/427) of lesions had the diagnosis upgraded to EGC after ESD. Furthermore, 8.9% (38/427) of lesions were diagnosed as submucosal invasive cancer.

To accept ESD as a treatment of EGC, the risk of lymph node metastasis must be absent. However, the presence of lymph node metastasis can be confirmed after surgical gastrectomy with lymph node dissection. EGC is a gastric cancer limited to the mucosa or submucosa, irrespective of the presence of lymph node metastasis. Submucosal and lymphovascular invasions are independent risk factors for lymph node metastases [11, 12]. Therefore, these findings are critical prognostic factors in patients with EGC. Submucosal invasion has been reported as an independent risk factor for lymphovascular invasion in endoscopically resected EGC, and the incidence of lymph node metastasis is significantly higher in submucosal invasive EGC [13]. This can be explained by the particular distribution of lymph capillaries in the mucosal layer. Although lymph capillaries are found in the deep lamina propria adjacent to and within the muscularis mucosa, most large lymph vessels are located in the submucosa [14]. However, there is no way to precisely evaluate submucosal or lymphovascular invasion before ESD. Recent reports examining the long-term outcomes of endoscopic resection for EGC (differentiated-type adenocarcinoma; no surface ulceration; a diameter of ≤2 cm; limited to the mucosa) showed comparable overall survival with surgery [15, 16]. For the endoscopic treatment of EGC, Japanese [17] and South Korean [18] gastric cancer treatment guidelines are almost the same. According to the guidelines, ESD is indicated as a standard treatment for lesions meeting the following criteria (absolute indications): (1) lesions limited to the mucosal layer, (2) well and/or moderately differentiated adenocarcinomas, (3) tumors ≤2 cm in length, (4) absence of ulceration or ulcer scar tissue, and (5) tumors without lymphovascular involvement.

In the present study, risk factors associated with EGC after ESD for HGD lesions were central depression, nodular surface, surface redness, large tumor size (≥1 cm), and tumor location in the upper third of the stomach. Additionally, associated factors with submucosal invasive EGC were nodular surface, submucosal fibrosis, tumor location in the upper third of the stomach, and larger tumor size (≥10 mm). As lesions progress, structural changes appear. Central depression and a nodular surface are associated with lesion progression [19]. Surface redness is associated with the development of vascular structures with disease progression [3]. Large tumor size is a known risk factor for EGC in adenoma, which can be understood as the size increases with disease progression [5]. However, we do not definitely know why the location of a lesion in the upper third of the stomach was a significant risk factor associated with EGC. Before now, some studies have shown that ESD is more difficult to treat and causes more complications if the lesion is located in the upper portion of the stomach [20]. A possible explanation for this is the technical factors associated with the endoscopic experience. EGC located in the lower third of the stomach, especially in the antrum, might be easily detected, but to detect EGC in the upper third of the stomach, more practical experience with endoscopic procedures might be required. Therefore, EGC lesions located in the upper third of the stomach might have a delayed or missed diagnosis. In addition, targeted biopsy of lesions located in the upper third of the stomach is difficult. Further studies might be required to clearly explain the reasons for this finding. Furthermore, we found that 38 submucosal invasive cancers were wrongly diagnosed as HGD in this study (8.9%, 38/427). Risk factors associated with submucosal invasive cancer were submucosal fibrosis, large tumor size, and tumor location in the upper third of the stomach. Adenocarcinomas were more closely related to submucosal fibrosis than were adenomatous lesions. A previous study reported that fibroblasts and myofibroblasts cause fibrosis of the submucosa as a result of the desmoplastic response to cancers [21]. A previous study had shown that submucosal invasive cancer causes submucosal fibrosis [22], and our study also indicates that submucosal invasion is closely related to submucosal fibrosis. As described above, lesions located in the upper part of the stomach are difficult to find and to perform targeted biopsy. Differences in wall thickness according to the location in the stomach might be associated with these results. The whole wall thickness is thicker in the antrum than in the body and cardia, and the thickness of the submucosal layer decreases from the antrum to the cardia and the body [23]. Thus, an EGC located in the upper portion of the stomach might be a deep invasive cancer even though it is the same size.

There are several limitations to this study. First, it was retrospectively conducted in a single center. The sample size of lesions might be too small for supporting these risk factors definitely. However, the identified risk factors, including other studies so far, may be helpful for further studies and provide evidence for caution with endoscopic treatment of EGC. Second, we used the conventional endoscopic appearances of the lesion for the analysis. If recent diagnostic technologies such as IEE were used, more accurate diagnoses may have been given.

In summary, because gastric HGD is a precancerous lesion and has a high rate of diagnosis upgrading to EGC, it should be removed. Risk factors associated with EGC were central depression, nodular surface, surface redness, tumor location (upper third of the stomach), and large tumor size. Furthermore, if submucosal fibrosis is suspected, the probability of submucosal invasive cancer increases. Therefore, for lesions with these risk factors, physicians should be cautious before deciding to treat with ESD. Patients should also be informed of the risks and benefits of undergoing more invasive treatments such as surgical gastrectomy with lymph node dissection.
